# Supplementary material for: Binding Free Energy Analysis of Colicin D, E3 and E8 to Their Respective Cognate Immunity Proteins Using Computational Simulations
Source: Molecules. 2025 Mar 12;30(6):1277. doi: 10.3390/molecules30061277 (PMC11944403; doi:10.3390/molecules30061277)
Supplement: Supplementary file 1 [file molecules-30-01277-s001.zip › molecules-3479788-supplementary.pdf]

Binding free energy analysis of colicin D, E3 and E8 to their respective cognate immunity proteins using computational simulations.

Mahesh Koirala*[1,2] and Clifton K. Fagerquist*[1]

[1]Produce Safety & Microbiology, Western Regional Research Center, Agricultural Research Service, U.S. Department of Agriculture, Albany, CA, USA 94710

[2]Research Participation Program administered by the Oak Ridge Institute for Science and Education, U.S. Department of Energy. Oak Ridge, TN, USA

*Corresponding authors: Mahesh Koirala (mahesh.koirala@usda.gov); Clifton K. Fagerquist (clifton.fagerquist@usda.gov)

1. Sequence of Colicin and Immunity Proteins

>AWJ36142.1 colicin D immunity protein (plasmid) [Escherichia coli O103 str. RM8385]

MNKMAMIDLAKLFLASKITAIEFSERICVERRRLYGVKDLSPNILNCGEELFMAAERFEPDADRADYEID
DNGLKVEVRSILEKFKL

>AWJ36133.1 colicin-D (plasmid) [Escherichia coli O103 str. RM8385]

MSDYEGSGPTEGIDYGHSMVVWPSTGLISGGDVKPGGSSGIAPSMPPGWGDYSPQGIALVQSVLFPGIIR
RIILDKELEEGDWSGWSVSVHSPWGNEKVSAARTVLENGLRGGLPEPSRPAAVSFARLEPASGNEQKIIR
LMVTQQLEQVTDIPASQLPAAGNNVPVKYRLMDLMQNGTQYMAIIGGIPMTVPVVDAVPVPDRSRPGTNI
KDVYSAPVSPNLPDLVLSVGQMNTPVLSNPEIQEEGVIAETGNYVEAGYTMSSNNHDVIVRFPEGSGVSP
LYISTVEILDSNGLSQRQEAENKAKDDFRVKKEEAVARAEAEKAKAELFSKAGVNQPPVYTQEMMERANS
VMNEQGALVLNNTASSVQLAMTGTGVWTAAGDIAGNISKFFSNALEKVTIPEVSPLLMRISLGALWFHSE
EAGAGSDIVPGRNLEAMFSLSAQMLAGQGVVIEPGATSVNLPVRGQLINSNGQLALDLLKTGNESIPAAV
PVLNAVRDTATGLDKITLPAVVGAPSRTILVNPVPQPSVPTDTGNHQPVPVTPVHTGTEVKPVEMPVTTI
TPVSDVGGLRDFIYWRPDAAGTGVEAVYVMLNEPLDSGRFSRKQLDKKYKHAGDFGISDTKKNRETLTKF
RDAIEEHLSDKDTVEKGTYRREKGSKVYFNPNTMNVVIIKSNGEFLSGWKINPDADNGRIYLETGEL

>PCG41551.1 colicin E3 immunity protein [Escherichia coli] (N-terminal methionine removed, RM7788 O113:H21)

GLKLDLTWFDKSTEDFKGEEYSKDFGDDGSVMESLGVPFKDNVNNGCFDVIAEWVPLLQPYFNHQIDIS

DNEYFVSFDYRDGDW

>PCG41550.1 colicin E3 [Escherichia coli] (83 residues removed from N-terminus, RM7788 O113:H21)

VAAPVAFGFPALSTPGAGGLAVSISAGALSAAIADIMAALKGPFKFGLWGVALYGVL

PSQIAKDDPNMMSKIVTSLPADDITESPVSSLPLDKATVNVNVRVVDDVKDERQNISVVSGVPMSVPVVD

AKPTERPGVFTASIPGAPVLNISVNNSTPAVQTLSPGVTNNTDKDVRPAGFTQGGNTRDAVIRFPKDSGH

NAVYVSVSDVLSPDQVKQRQDEENRRQQEWDATHPVEAAERNYERARAELNQANEDVARNQERQAKAVQV

YNSRKSELDAANKTLADAIAEIKQFNRFAHDPMAGGHRMWQMAGLKAQRAQTDVNNKQAAFDAAAKEKSD

ADAALSSAMESRKKKEDKKRSAENNLNDEKNKPRKGFKDYGHDYHPAPKTENIKGLGDLKPGIPKTPKQN

GGGKRKRWTGDKGRKIYEWDSQHGELEGYRASDGQHLGSFDPKTGNQLKGPDPKRNIKKYL

>AWJ52141.1 colicin E8 immunity protein (plasmid) [Escherichia coli O43 str. RM10042]

MELKNSISDYTETEFKKIIEDIINCEGDEKKQDDNLEHFISVTEHPSGSDLIYYPEGNNDGSPEAVIKEI

KEWRAANGKSGFKQG

>AWJ52140.1 colicin (plasmid) [Escherichia coli O43 str. RM10042] (83 residues removed from N-terminus)

VAVPVAFGFPALSTPGAGGLAVSISAGALSAAIADIMAALKGPFKFGLWGVALYGVL

PSQIAKDDPNMMSKIVTSLPADDITESPVSSLPLDKATVNVNVRVVDDVKDERQNISVVSGVPMSVPVVD

AKPTERPGVFTASIPGAPVLNISVNNSTPAVQTLSPGVTNNTDKDVRPAGFTQGGNTRDAVIRFPKDSGH

NAVYVSVSDVLSPDQVKQRQDEENRRQQEWDATHPVEAAERNYERARAELNQANEDVARNQERQAKAVQV

YNSRKSELDAANKTLADAIAEIKQFDRFAHDPMSGGHRMWQMAGLKAQRAQTDVNNKQAAFDAAAKEKSD

ADAALSAAQERRKQKENKEKDAKDKLDKESKRNKPGKATGKGKPVGDKWLDDAGKDSGAPIPDRIADKLR

DKEFKNFDDFRRKFWEEVSKDPELSKQFNPGNKKRLSQGLAPRARNKDTVGGRRSFELHHDKPISQDGGV

YDMDNLRITTPKRHIDIHRGQ

2. Results of Molecular Dynamics (MD) Simulation

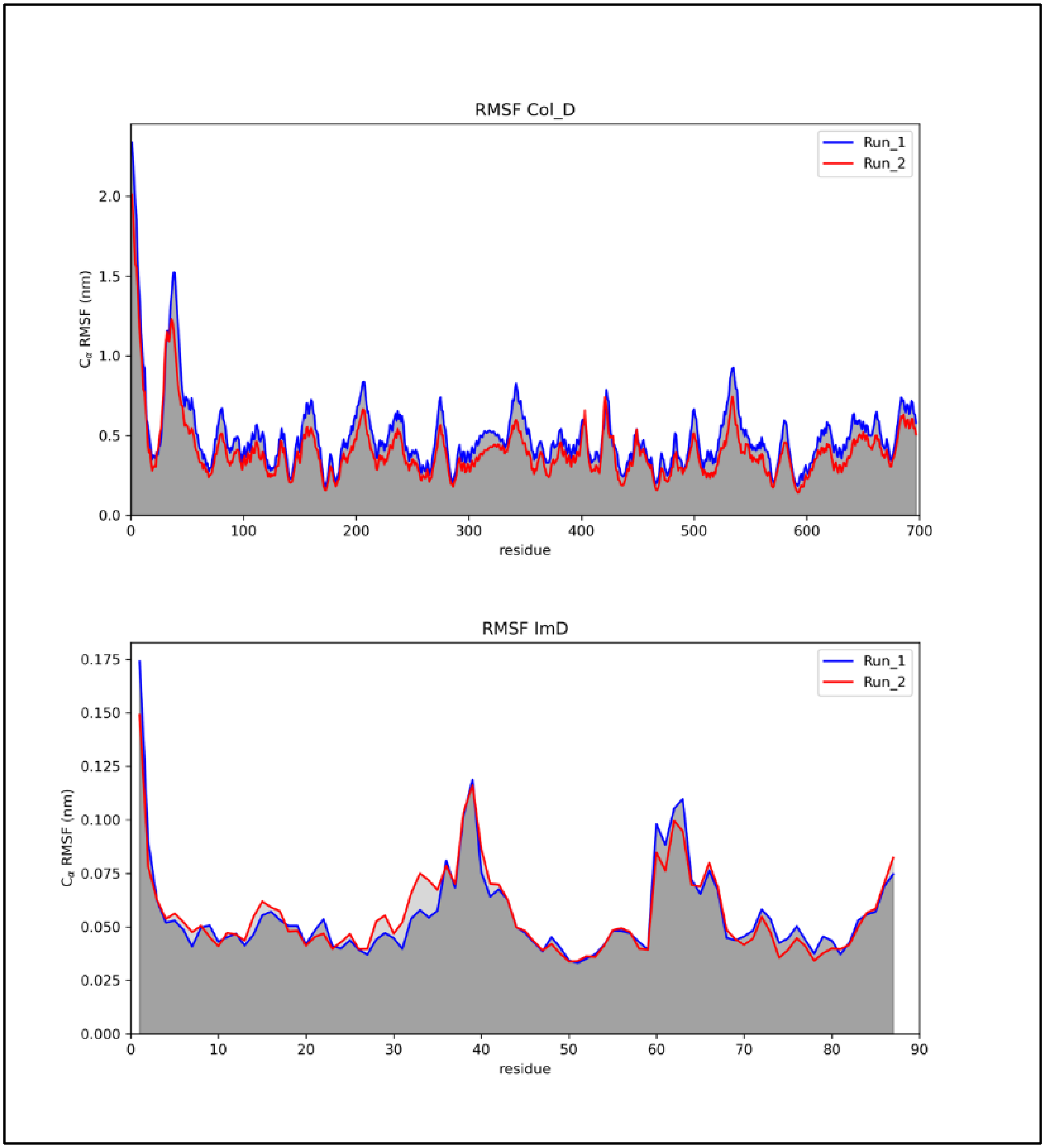

Figure S1. RMSF analysis of Col_D and ImD

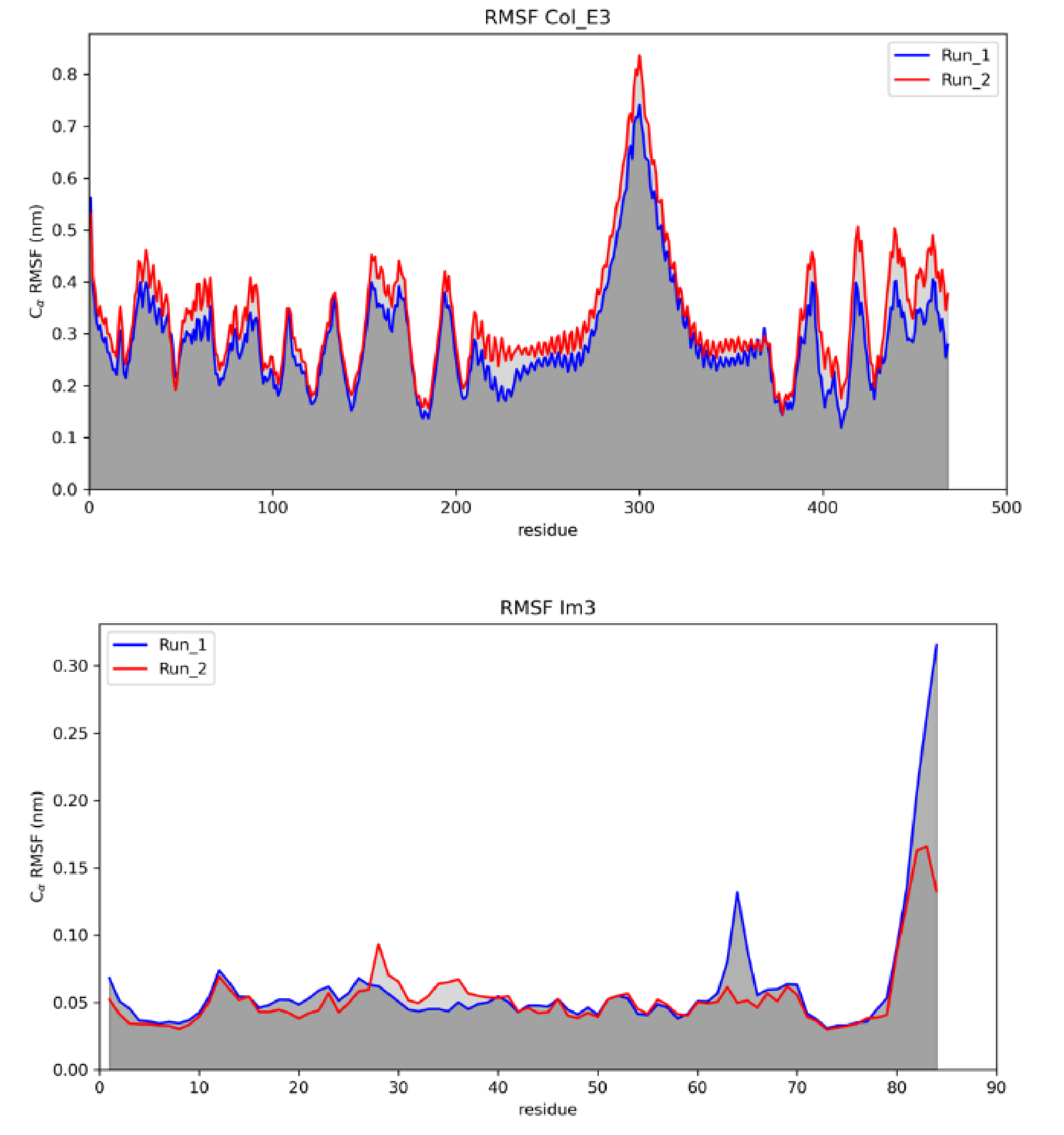

Figure S2. RMSF analysis of Col_E3 and Im3

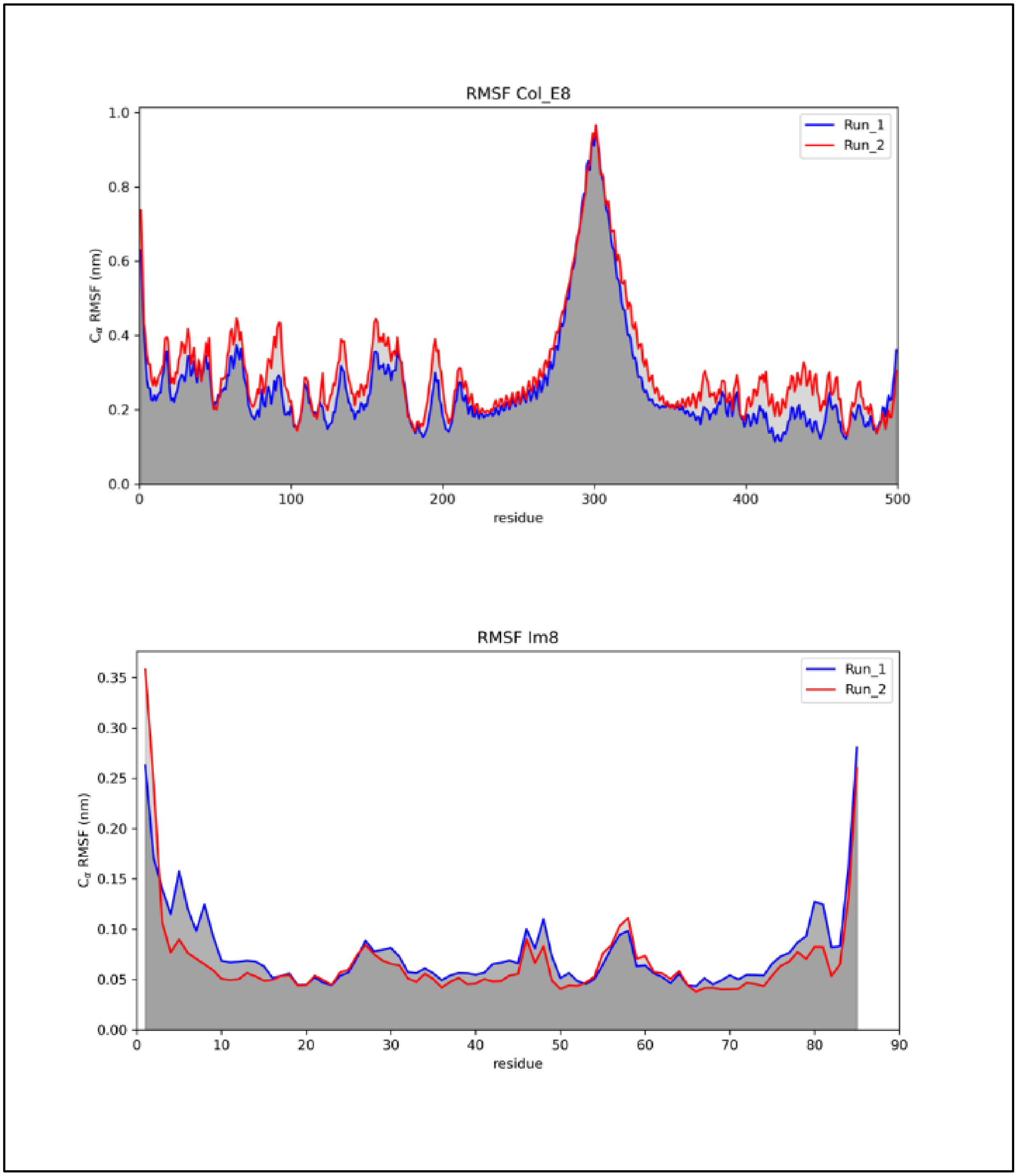

Figure S3. RMSF analysis of Col_E8 and Im8

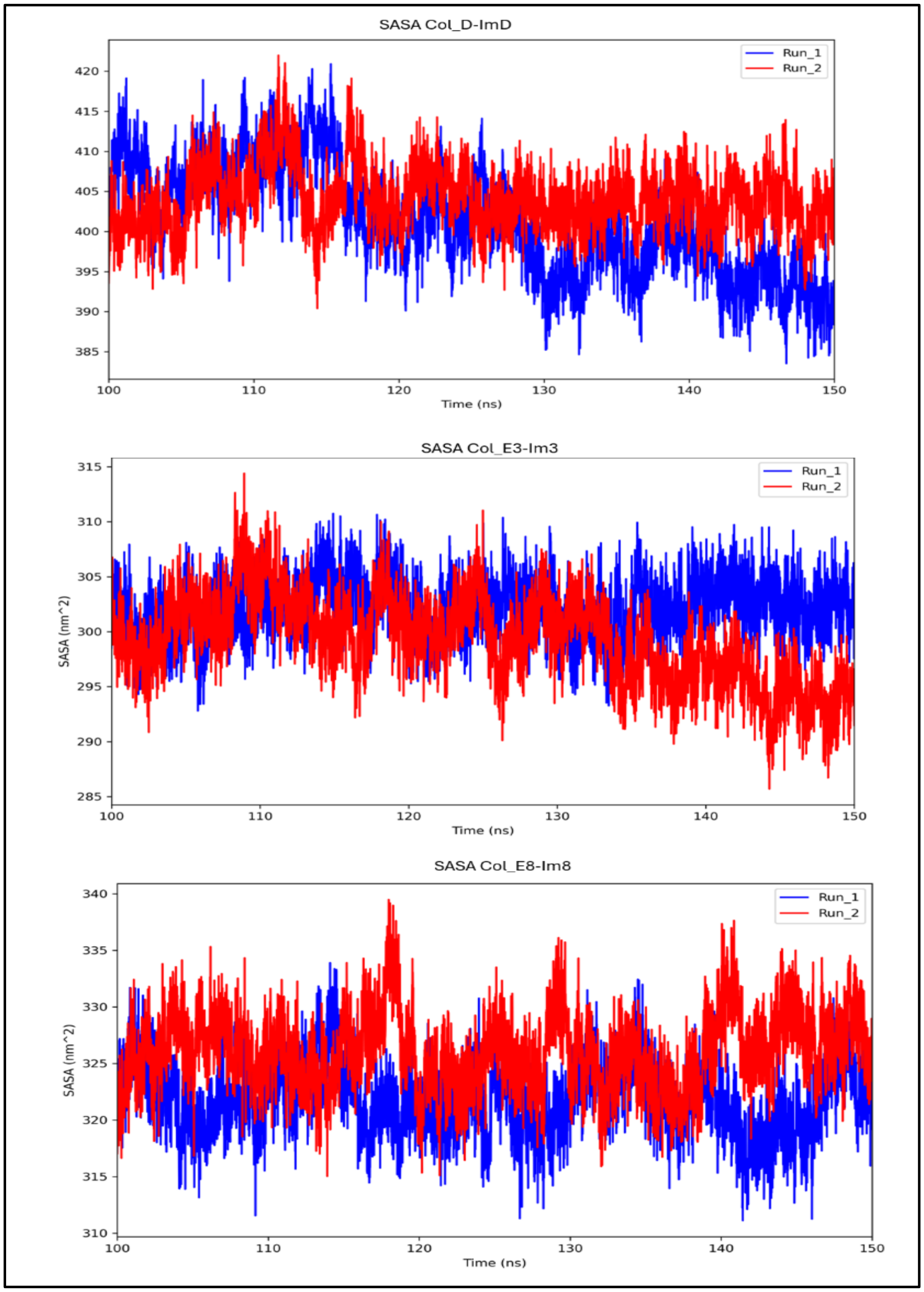

Figure S4. SASA of colicin/immunity complexes over 150ns, showing stability in the 100-150 ns windows used for free energy analysis.

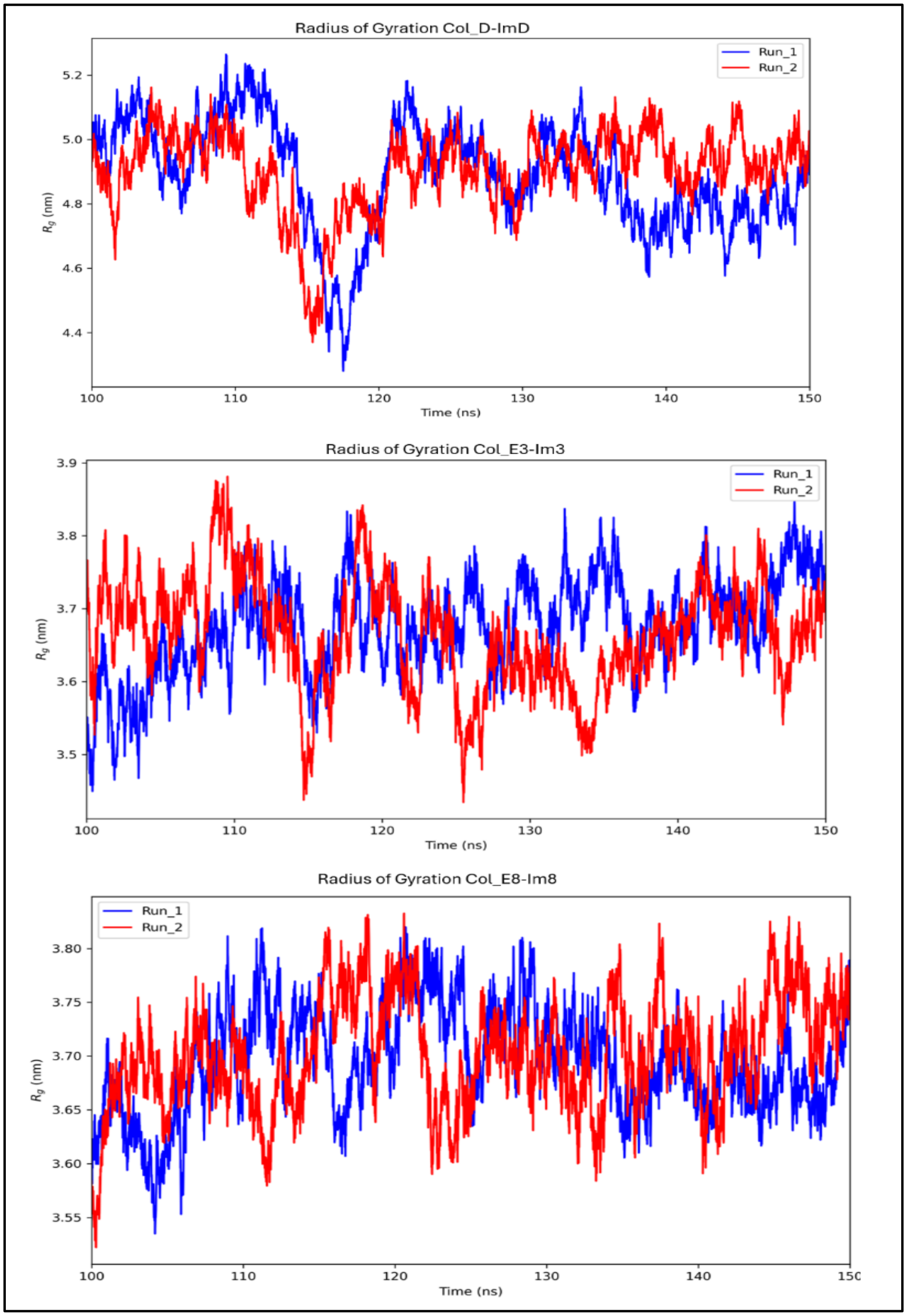

Figure S5. Radius of Gyration of colicin/immunity complexes over 150ns, showing stability in the 100-150 ns windows used for free energy analysis.

Table S1. Per-residue decomposition of the binding free energy for Col_D (chain B) in complex with ImD (chain A), obtained in Run1 using the MM-PBSA method with energy values given in kcal/mol.

Chain	Residue	van der Waals		Electrostatics		Polar Solvation		Non-Polar Solvation		Total	
		Avg.	SEM	Avg.	SEM	Avg.	SEM	Avg.	SEM	Avg.	SEM
A	ILE:21	-0.36	0.08	-1.43	0.11	1.38	0.07	0	0	-0.41	0.23
A	SER:24	-0.64	0.07	-1.53	0.16	2.37	0.09	0	0	0.2	0.24
A	GLU:25	-1.15	0.07	0.48	0.66	1.08	0.61	0	0	0.41	0.91
A	ILE:27	-0.2	0.09	2.86	0.07	-2.73	0.02	0	0	-0.07	0.21
A	CYS:28	-2.03	0.07	2.68	0.09	-2.02	0.05	0	0	-1.37	0.18
A	ARG:31	-1.9	0.1	-52.45	0.38	53.12	0.27	0	0	-1.23	0.54
A	ARG:32	-2.55	0.1	-72.74	0.36	68.57	0.26	0	0	-6.71	0.52
A	LEU:34	-0.17	0.07	1.19	0.09	-1.08	0.04	0	0	-0.06	0.21
A	TYR:35	-3.55	0.1	-4.47	0.08	5.43	0.04	0	0	-2.58	0.25
A	GLY:36	-0.35	0.04	-2.24	0.15	2.48	0.12	0	0	-0.1	0.22
A	LEU:40	-0.19	0.07	0.51	0.1	-0.52	0.07	0	0	-0.19	0.23
A	PRO:42	-0.29	0.06	-0.3	0.09	0.42	0.04	0	0	-0.17	0.21
A	LEU:45	-1.84	0.07	-2.32	0.08	2.48	0.04	0	0	-1.68	0.21
A	ASN:46	-1.47	0.07	-6.8	0.16	7.69	0.12	0	0	-0.58	0.28
A	GLU:49	-1.78	0.1	30.28	0.36	-26.15	0.31	0	0	2.35	0.51
A	GLU:50	-0.48	0.09	22.7	1.11	-21.15	1.05	0	0	1.07	1.54
A	PHE:52	-2.48	0.11	-5.2	0.09	5.24	0.05	0	0	-2.43	0.24
A	MET:53	-2.42	0.07	-3.31	0.09	3.39	0.07	0	0	-2.35	0.22
A	ALA:55	-0.48	0.05	-5.66	0.12	6.07	0.05	0	0	-0.07	0.19
A	GLU:56	-0.16	0.1	-69.7	0.47	69.7	0.31	0	0	-0.16	0.6
A	ARG:57	-1.82	0.11	-1.96	0.66	4.46	0.47	0	0	0.69	0.86
A	PHE:58	-0.59	0.11	-7.14	0.11	7.93	0.08	0	0	0.2	0.26
A	GLU:59	0.87	0.09	-56.86	0.75	54	0.61	0	0	-1.99	0.99
A	PRO:60	-0.85	0.06	2.66	0.11	-2.23	0.09	0	0	-0.42	0.23
A	ASP:61	-0.56	0.1	-25.03	0.6	26.38	0.54	0	0	0.79	0.83
A	ALA:62	-1.39	0.06	-0.46	0.13	0.48	0.09	0	0	-1.37	0.22
A	ASP:63	-1.88	0.1	3.78	0.47	0.65	0.47	0	0	2.54	0.69
A	ARG:64	-0.46	0.1	-7.23	0.43	6.9	0.36	0	0	-0.79	0.62
A	ALA:65	-0.31	0.06	-0.81	0.15	0.77	0.09	0	0	-0.34	0.23
A	ASP:66	-0.23	0.07	21.67	0.44	-21.06	0.43	0	0	0.38	0.64
A	TYR:67	-3.45	0.11	-8.09	0.26	7.93	0.12	0	0	-3.61	0.36
A	GLU:68	0.21	0.08	-38.41	0.71	38.96	0.64	0	0	0.76	0.97
B	SER:470	-0.07	0.08	-2.51	0.2	2.33	0.15	0	0	-0.24	0.3
B	ASN:471	-0.31	0.08	-2.46	0.26	2.35	0.23	0	0	-0.42	0.38
B	VAL:557	-0.13	0.09	-1.96	0.08	1.87	0.05	0	0	-0.22	0.21
B	THR:558	-1.44	0.08	-4.82	0.29	5.35	0.18	0	0	-0.92	0.38
B	THR:559	-0.54	0.09	-17.95	0.2	13.25	0.13	0	0	-5.23	0.3
B	ILE:560	-1.45	0.09	-7.46	0.11	5.29	0.06	0	0	-3.62	0.23
B	THR:561	-0.58	0.08	-5.47	0.21	4.06	0.15	0	0	-1.98	0.32
B	LYS:603	0.48	0.1	-128.75	1.26	121.72	1.1	0	0	-6.56	1.68
B	LYS:607	0.52	0.09	-162.67	0.65	152.26	0.55	0	0	-9.9	0.88
B	LYS:608	0.45	0.09	-142.96	0.42	134.49	0.36	0	0	-8.01	0.58
B	TYR:609	-0.41	0.13	-5.95	0.14	5.73	0.07	0	0	-0.63	0.28
B	LYS:610	-3.65	0.09	-118.35	0.53	118.29	0.45	0	0	-3.7	0.73
B	HIS:611	-2.31	0.07	-19.17	0.12	19.9	0.07	0	0	-1.57	0.23
B	ALA:612	-0.43	0.07	-1.65	0.08	1.68	0.03	0	0	-0.4	0.17
B	GLY:613	-1.18	0.05	1.7	0.07	-0.53	0.04	0	0	-0.01	0.15
B	ASP:614	-0.7	0.08	-14.1	0.31	17.59	0.28	0	0	2.8	0.45
B	SER:618	-0.07	0.06	0.27	0.23	-0.26	0.13	0	0	-0.06	0.31
B	LYS:622	0.13	0.09	-75.88	0.75	73.7	0.66	0	0	-2.05	1.02
B	TYR:649	-1.34	0.1	-3.07	0.12	4.67	0.08	0	0	0.27	0.25
B	ARG:650	-0.25	0.1	-38.45	0.57	37.71	0.5	0	0	-0.99	0.8
B	ARG:651	-3.73	0.07	-56.06	0.39	58.8	0.35	0	0	-0.99	0.58
B	GLU:652	-0.33	0.09	60	0.68	-58.02	0.62	0	0	1.65	0.94
B	MET:664	-0.56	0.08	0.07	0.08	-0.23	0.03	0	0	-0.72	0.21
B	ILE:668	-0.15	0.09	0.47	0.07	-0.44	0.02	0	0	-0.12	0.2
B	LEU:676	-0.64	0.07	3.34	0.11	-3.08	0.05	0	0	-0.38	0.23
B	SER:677	0.27	0.07	-11.74	0.1	7.88	0.05	0	0	-3.59	0.18
B	GLY:678	-0.2	0.05	1.5	0.06	-1.28	0.04	0	0	0.01	0.13
B	TRP:679	-3.7	0.1	5.54	0.11	-2.91	0.05	0	0	-1.07	0.27
B	LYS:680	-1.2	0.09	-12.27	0.33	9.26	0.28	0	0	-4.22	0.48
B	ILE:681	-0.29	0.09	-6.3	0.07	3.8	0.03	0	0	-2.8	0.21
B	ASN:682	-3.21	0.09	-6.62	0.13	6.81	0.06	0	0	-3.02	0.22
B	PRO:683	-0.83	0.07	0.63	0.13	-0.53	0.06	0	0	-0.73	0.22
B	ASP:684	-1.09	0.07	-13.67	0.87	16.39	0.84	0	0	1.62	1.22
B	ALA:685	-1.69	0.05	-2.75	0.09	2.68	0.05	0	0	-1.77	0.18
B	ASP:686	-1.06	0.08	11.47	0.4	-9.87	0.4	0	0	0.54	0.59
B	ASN:687	-1.47	0.07	-5.94	0.15	6.62	0.12	0	0	-0.78	0.26
B	GLY:688	-0.2	0.05	-0.09	0.08	0.31	0.05	0	0	0.02	0.15
B	ARG:689	-0.26	0.08	-8.06	0.42	7.91	0.37	0	0	-0.41	0.61
B	LEU:697	-0.31	0.09	43.95	0.41	-42.7	0.39	0	0	0.94	0.59

*SEM: Standard Error of Mean

Table S2. Per-residue decomposition of the binding free energy for Col_D (chain B) in complex with ImD (chain A), obtained in Run2 using the MM-PBSA method with energy values given in kcal/mol.

Chain	Residue	van der Waals		Electrostatics		Polar Solvation		Non-Polar Solvation		Total	
		Avg.	SEM	Avg.	SEM	Avg.	SEM	Avg.	SEM	Avg.	SEM
A	SER:24	-0.76	0.07	-1.19	0.11	2.01	0.08	0	0	0.06	0.2
A	GLU:25	-1.45	0.06	-0.83	0.49	1.96	0.48	0	0	-0.31	0.71
A	CYS:28	-2.05	0.07	2.54	0.1	-1.81	0.06	0	0	-1.33	0.19
A	ARG:31	-1.87	0.1	-51.29	0.34	51.71	0.23	0	0	-1.46	0.49
A	ARG:32	-2.32	0.09	-69.32	0.36	64.99	0.24	0	0	-6.64	0.51
A	LEU:34	-0.17	0.08	1.24	0.09	-1.17	0.05	0	0	-0.1	0.22
A	TYR:35	-3.52	0.12	-4.08	0.08	5.22	0.06	0	0	-2.39	0.26
A	GLY:36	-0.32	0.04	-1.94	0.15	2.19	0.12	0	0	-0.06	0.23
A	VAL:37	-0.2	0.07	3.02	0.11	-2.62	0.11	0	0	0.21	0.23
A	LYS:38	0.1	0.07	-69.19	0.84	67.55	0.79	0	0	-1.55	1.17
A	LEU:40	-0.14	0.07	1.03	0.09	-1	0.06	0	0	-0.11	0.21
A	PRO:42	-0.32	0.06	-0.73	0.08	0.87	0.04	0	0	-0.18	0.21
A	LEU:45	-1.94	0.07	-2.76	0.09	2.97	0.04	0	0	-1.73	0.22
A	ASN:46	-1.44	0.08	-7.54	0.15	8.23	0.11	0	0	-0.75	0.26
A	GLU:49	-2.02	0.09	29.1	0.31	-24.79	0.26	0	0	2.29	0.44
A	GLU:50	-0.56	0.08	22.72	1.01	-21.3	0.96	0	0	0.86	1.41
A	PHE:52	-2.5	0.11	-5.18	0.09	5.27	0.06	0	0	-2.4	0.25
A	MET:53	-2.49	0.08	-2.95	0.09	3.11	0.06	0	0	-2.33	0.22
A	ALA:55	-0.46	0.05	-5.22	0.09	5.9	0.06	0	0	0.21	0.18
A	GLU:56	-0.48	0.09	-66.57	0.47	69.63	0.32	0	0	2.58	0.61
A	ARG:57	-1.77	0.11	-2.62	0.73	4.95	0.49	0	0	0.57	0.92
A	PHE:58	-0.41	0.11	-5.89	0.09	6.36	0.08	0	0	0.06	0.27
A	GLU:59	0.7	0.1	-44.14	0.46	43.56	0.4	0	0	0.12	0.64
A	PRO:60	-0.34	0.06	2.91	0.08	-2.8	0.07	0	0	-0.23	0.19
A	ASP:61	0.05	0.09	-23.97	0.52	24.45	0.52	0	0	0.53	0.76
A	ALA:62	-0.32	0.04	-0.2	0.11	0.43	0.08	0	0	-0.09	0.2
A	ASP:63	-0.68	0.07	15.4	0.34	-13.43	0.34	0	0	1.29	0.51
A	ARG:64	-0.22	0.09	-8.44	0.4	8.09	0.32	0	0	-0.57	0.58
A	ASP:66	-0.2	0.07	17.4	0.52	-16.9	0.52	0	0	0.3	0.76
A	TYR:67	-3.07	0.12	-10.69	0.21	8.85	0.11	0	0	-4.91	0.32
A	GLU:68	-0.52	0.07	-12.39	0.4	14.48	0.39	0	0	1.56	0.58
B	ILE:560	-1.19	0.09	0.67	0.16	-0.33	0.13	0	0	-0.85	0.29
B	THR:561	-0.11	0.09	3.06	0.21	-2.86	0.18	0	0	0.09	0.34
B	LYS:603	-0.17	0.1	-69.09	0.92	67.27	0.89	0	0	-1.99	1.29
B	GLN:604	-0.23	0.09	6.21	0.2	-6.04	0.17	0	0	-0.06	0.33
B	LYS:607	1.2	0.09	-163.24	0.57	151.48	0.49	0	0	-10.56	0.78
B	LYS:608	0.11	0.08	-144.82	0.41	134.91	0.36	0	0	-9.8	0.58
B	LYS:610	-3.79	0.08	-116.56	0.46	116.85	0.42	0	0	-3.51	0.65
B	HIS:611	-2.31	0.07	-19.44	0.12	20.09	0.07	0	0	-1.66	0.23
B	ALA:612	-0.42	0.06	-1.69	0.08	1.71	0.03	0	0	-0.39	0.17
B	GLY:613	-1.2	0.04	1.65	0.07	-0.52	0.04	0	0	-0.07	0.15
B	ASP:614	-0.59	0.09	-15.15	0.3	19.13	0.28	0	0	3.38	0.44
B	LYS:622	-0.13	0.08	-73.42	0.58	72.06	0.53	0	0	-1.49	0.81
B	TYR:649	-1.3	0.11	-3	0.14	4.3	0.09	0	0	0.01	0.28
B	ARG:650	-0.25	0.1	-38.61	0.42	37.83	0.4	0	0	-1.03	0.64
B	ARG:651	-3.85	0.07	-56.57	0.32	59.17	0.3	0	0	-1.25	0.53
B	GLU:652	-0.09	0.09	61.55	0.52	-60.26	0.5	0	0	1.2	0.74
B	LYS:653	-0.09	0.06	-46.58	0.52	45.84	0.51	0	0	-0.83	0.76
B	MET:664	-0.54	0.08	0.12	0.08	-0.29	0.03	0	0	-0.71	0.21
B	ILE:668	-0.15	0.08	0.46	0.07	-0.43	0.02	0	0	-0.12	0.19
B	LYS:670	-0.14	0.1	-73.13	0.7	70.64	0.57	0	0	-2.64	0.93
B	LEU:676	-0.62	0.07	3.22	0.11	-2.95	0.05	0	0	-0.35	0.22
B	SER:677	0.23	0.07	-12.1	0.1	8.17	0.05	0	0	-3.71	0.19
B	GLY:678	-0.21	0.04	1.31	0.06	-1.15	0.04	0	0	-0.05	0.13
B	TRP:679	-3.73	0.1	5.54	0.12	-2.92	0.05	0	0	-1.11	0.26
B	LYS:680	-1.2	0.09	-11.6	0.35	8.35	0.28	0	0	-4.45	0.49
B	ILE:681	-0.45	0.09	-6.32	0.08	3.96	0.03	0	0	-2.81	0.21
B	ASN:682	-3.33	0.09	-6.2	0.14	6.65	0.07	0	0	-2.88	0.24
B	PRO:683	-0.81	0.06	0.78	0.14	-0.68	0.08	0	0	-0.72	0.23
B	ASP:684	-1.13	0.07	-11.28	0.75	13.56	0.76	0	0	1.15	1.08
B	ALA:685	-1.72	0.05	-2.83	0.12	2.76	0.06	0	0	-1.79	0.19
B	ASP:686	-1.02	0.08	10.89	0.41	-9.49	0.39	0	0	0.38	0.59
B	ASN:687	-1.72	0.07	-5.32	0.16	6.31	0.12	0	0	-0.73	0.26
B	GLY:688	-0.19	0.05	0.1	0.08	0.08	0.05	0	0	-0.02	0.16
B	LEU:697	-0.32	0.08	44.68	0.39	-43.29	0.37	0	0	1.07	0.57

*SEM: Standard Error of Mean

Table S3. Per-residue decomposition of the binding free energy for Col_E3 (chain B) in complex with Im3 (chain A), obtained in Run1 using the MM-PBSA method with energy values given in kcal/mol.

Chain	Residue	van der Waals		Electrostatics		Polar Solvation		Non-Ploar Solvation		Total	
		Avg.	SEM	Avg.	SEM	Avg.	SEM	Avg.	SEM	Avg.	SEM
A	LEU:2	-0.73	0.02	0.84	0.03	-0.89	0.01	0	0	-0.78	0.07
A	ASP:5	0.59	0.03	-120.5	0.16	118.57	0.16	0	0	-1.33	0.23
A	THR:7	-0.71	0.02	-0.2	0.03	0.65	0.02	0	0	-0.25	0.06
A	PHE:9	-4.06	0.03	1	0.03	-0.35	0.01	0	0	-3.41	0.07
A	ASP:10	-0.22	0.03	-63.64	0.14	63.74	0.12	0	0	-0.12	0.19
A	GLU:14	-0.82	0.02	-100.22	0.15	100.49	0.14	0	0	-0.55	0.21
A	ASP:15	0.01	0.03	-92.63	0.29	91.48	0.26	0	0	-1.14	0.39
A	PHE:16	-3.09	0.03	0.72	0.04	0.26	0.03	0	0	-2.11	0.09
A	GLU:19	-0.41	0.02	-82.24	0.21	82.86	0.2	0	0	0.2	0.29
A	TYR:21	-0.75	0.04	-0.7	0.07	0.86	0.05	0	0	-0.59	0.11
A	GLY:26	-0.17	0.02	-0.81	0.03	1.35	0.02	0	0	0.37	0.06
A	ASP:27	-0.8	0.02	-66.36	0.19	66.64	0.17	0	0	-0.53	0.26
A	ASP:28	-0.74	0.03	-46.9	0.1	47.68	0.09	0	0	0.05	0.15
A	GLY:29	-0.9	0.02	-0.07	0.03	0.55	0.02	0	0	-0.42	0.05
A	SER:30	-0.55	0.03	0.69	0.05	-0.12	0.02	0	0	0.01	0.07
A	MET:32	-0.97	0.02	-0.01	0.02	0.05	0.01	0	0	-0.93	0.06
A	GLU:33	-1.53	0.02	-34.46	0.09	36.74	0.09	0	0	0.76	0.14
A	SER:34	-1.98	0.02	0.71	0.03	2.28	0.03	0	0	1.02	0.06
A	LEU:35	-0.93	0.02	-0.91	0.03	1.17	0.01	0	0	-0.67	0.06
A	GLY:36	-1.69	0.01	-2.06	0.03	2.87	0.02	0	0	-0.88	0.05
A	VAL:37	-1.08	0.02	0.86	0.02	-0.35	0.01	0	0	-0.56	0.06
A	PRO:38	-3.31	0.02	-5.2	0.02	5.64	0.01	0	0	-2.88	0.06
A	PHE:39	-4.48	0.03	-3.5	0.03	5.4	0.02	0	0	-2.58	0.07
A	LYS:40	-3.87	0.03	36.46	0.12	-33.47	0.1	0	0	-0.88	0.17
A	ASP:41	-2.32	0.03	-58.29	0.13	61.23	0.11	0	0	0.62	0.18
A	ASN:42	-1.45	0.02	-11.14	0.04	12.12	0.02	0	0	-0.47	0.07
A	VAL:43	-3.05	0.02	1.25	0.02	1.78	0.01	0	0	-0.02	0.06
A	ASN:44	-3.96	0.02	-5.81	0.04	8.05	0.02	0	0	-1.71	0.07
A	ASN:45	-3.94	0.03	-7.39	0.05	8.67	0.03	0	0	-2.66	0.08
A	GLY:46	-2.16	0.02	1.01	0.03	1.14	0.02	0	0	-0.01	0.06
A	CYS:47	-2.86	0.02	-7.08	0.03	6.08	0.02	0	0	-3.86	0.06
A	PHE:48	-2.57	0.03	-1	0.03	1.17	0.02	0	0	-2.4	0.08
A	ASP:49	0.14	0.03	-97.21	0.22	96.33	0.2	0	0	-0.73	0.3
A	ILE:51	-0.4	0.02	0.28	0.03	-0.21	0.01	0	0	-0.33	0.07
A	ALA:52	-1.18	0.02	1.28	0.02	-0.83	0.01	0	0	-0.73	0.05
A	GLU:53	-3.28	0.02	-38.71	0.12	44.87	0.11	0	0	2.88	0.18
A	VAL:55	-0.65	0.03	-0.01	0.02	0.03	0.01	0	0	-0.63	0.06
A	PRO:56	-3.6	0.02	-2.67	0.02	2.62	0.01	0	0	-3.66	0.06
A	LEU:57	-3.3	0.02	0.5	0.02	0.52	0.01	0	0	-2.28	0.06
A	GLN:59	-2.21	0.03	-1.65	0.04	2.62	0.02	0	0	-1.24	0.08
A	PRO:60	-3.17	0.02	-0.67	0.02	0.7	0.01	0	0	-3.14	0.06
A	TYR:61	-1.35	0.03	-0.88	0.03	2.08	0.02	0	0	-0.15	0.08
A	PHE:62	-0.25	0.03	0.5	0.04	-0.42	0.03	0	0	-0.17	0.08
A	ASN:63	-0.39	0.02	-3.15	0.11	3.14	0.09	0	0	-0.4	0.16
A	HIS:64	-0.4	0.03	0.33	0.07	-0.12	0.07	0	0	-0.18	0.11
A	GLN:65	-2.04	0.02	-2.74	0.08	3.48	0.06	0	0	-1.3	0.12
A	ILE:66	-0.33	0.03	1.14	0.03	-1.09	0.02	0	0	-0.28	0.07
A	ILE:68	-0.29	0.03	-0.41	0.04	0.38	0.02	0	0	-0.33	0.07
A	GLU:72	-0.53	0.03	-69.07	0.21	68.77	0.19	0	0	-0.83	0.29
A	PHE:74	-3	0.03	0.77	0.03	0.11	0.01	0	0	-2.11	0.07
A	SER:76	-0.63	0.02	0.28	0.04	0.18	0.02	0	0	-0.18	0.06
A	ASP:78	-0.55	0.03	-86.06	0.17	85.9	0.15	0	0	-0.71	0.23
A	TYR:79	-3.66	0.04	-1.04	0.05	3.14	0.03	0	0	-1.56	0.09
A	ARG:80	-2.75	0.03	76.4	0.26	-73.72	0.21	0	0	-0.07	0.34
A	ASP:81	0.02	0.02	-65.66	0.22	64.9	0.2	0	0	-0.74	0.3
A	GLY:82	-0.07	0.02	-2.09	0.06	2.06	0.04	0	0	-0.1	0.09
A	TRP:84	-1.05	0.04	-76.37	0.33	75.87	0.33	0	0	-1.55	0.47
B	PHE:46	-0.82	0.03	-2.81	0.03	2.99	0.02	0	0	-0.65	0.08
B	GLY:47	-0.41	0.01	-2.58	0.03	2.76	0.02	0	0	-0.23	0.06
B	LEU:48	-0.62	0.02	-0.2	0.02	0.2	0.02	0	0	-0.62	0.06
B	ASP:92	-0.23	0.02	82.42	0.2	-80.98	0.2	0	0	1.21	0.29
B	LYS:93	-1.05	0.02	-100.8	0.21	101.69	0.2	0	0	-0.16	0.29
B	ALA:94	-0.62	0.02	-2.57	0.03	2.28	0.02	0	0	-0.91	0.06
B	THR:95	-1.61	0.02	-1.07	0.05	1.51	0.03	0	0	-1.17	0.08
B	ASN:97	-0.8	0.02	-0.24	0.08	0.86	0.06	0	0	-0.18	0.11
B	VAL:98	-0.2	0.02	1.48	0.03	-1.41	0.02	0	0	-0.13	0.06
B	ARG:101	-1.71	0.03	-97.97	0.1	100.59	0.08	0	0	0.92	0.15
B	VAL:119	-1.74	0.03	-3.18	0.02	3.17	0.02	0	0	-1.75	0.07
B	PRO:120	-2.75	0.02	1.3	0.02	2.28	0.02	0	0	0.83	0.06
B	MET:121	-2.49	0.02	-2.16	0.02	2.9	0.01	0	0	-1.75	0.06
B	SER:122	-2.55	0.02	-0.53	0.05	2.5	0.02	0	0	-0.58	0.07
B	VAL:123	-0.83	0.03	0.06	0.03	0.09	0.01	0	0	-0.68	0.07
B	PRO:124	-0.66	0.02	-2.41	0.02	2.36	0.01	0	0	-0.71	0.06

B	PRO:142	-0.4	0.02	-1.32	0.02	1.37	0.02	0	0	-0.35	0.06
B	PHE:178	-2.53	0.03	1.91	0.05	-1.07	0.04	0	0	-1.69	0.09
B	THR:179	-0.34	0.03	2.86	0.1	-1.96	0.03	0	0	0.55	0.13
B	GLN:180	-0.41	0.02	3.31	0.06	-2.55	0.04	0	0	0.36	0.09
B	GLY:181	-0.63	0.02	1.84	0.03	-0.14	0.01	0	0	1.07	0.05
B	GLY:182	-0.9	0.02	0.09	0.04	0.07	0.02	0	0	-0.74	0.06
B	ASN:183	-0.2	0.03	1.31	0.07	-1.3	0.05	0	0	-0.19	0.1
B	THR:184	-0.31	0.03	0.85	0.04	-0.7	0.01	0	0	-0.16	0.07
B	ARG:185	-1.85	0.03	-104.99	0.13	104.51	0.11	0	0	-2.33	0.19
B	ASP:186	-0.36	0.04	92.48	0.09	-88.93	0.07	0	0	3.2	0.13
B	ARG:216	-0.14	0.03	-93.04	0.16	90.79	0.13	0	0	-2.39	0.22
B	ARG:223	-0.8	0.03	-120.45	0.18	118.99	0.15	0	0	-2.26	0.25
B	GLU:226	-0.7	0.02	98.31	0.2	-95.62	0.2	0	0	1.98	0.29
B	TRP:227	-2.95	0.03	0.16	0.03	0.72	0.02	0	0	-2.07	0.09
B	THR:230	-1.86	0.02	0.47	0.03	2.27	0.02	0	0	0.88	0.07
B	HIS:231	-1.73	0.03	1.96	0.04	1.15	0.04	0	0	1.38	0.09
B	LYS:372	-0.34	0.02	-85.05	0.17	84.07	0.15	0	0	-1.31	0.24
B	GLY:373	-0.31	0.02	-1.55	0.04	1.55	0.02	0	0	-0.31	0.06
B	PHE:374	-7.51	0.04	-1.8	0.03	4.91	0.02	0	0	-4.41	0.08
B	LYS:375	-1.46	0.02	-141.97	0.27	141.23	0.26	0	0	-2.2	0.38
B	TYR:377	-4.5	0.03	-0.44	0.05	3.32	0.04	0	0	-1.61	0.09
B	GLY:378	-1.66	0.01	-1.84	0.03	2.85	0.01	0	0	-0.66	0.05
B	HIS:379	-0.7	0.02	-1.08	0.04	1.15	0.04	0	0	-0.63	0.08
B	ASP:380	-0.61	0.03	92.24	0.22	-88.65	0.21	0	0	2.98	0.31
B	TYR:381	-3.27	0.04	-8.73	0.04	6.04	0.03	0	0	-5.96	0.09
B	HIS:382	-0.82	0.03	3.87	0.05	-2.5	0.04	0	0	0.55	0.09
B	PRO:383	-3.1	0.02	-3.71	0.02	4.4	0.02	0	0	-2.41	0.06
B	ALA:384	-0.39	0.02	-5.41	0.04	3.39	0.03	0	0	-2.41	0.07
B	LYS:386	-1.7	0.03	-178.97	0.24	179.55	0.21	0	0	-1.12	0.32
B	THR:387	-0.3	0.02	-6.08	0.06	6.17	0.04	0	0	-0.22	0.09
B	ASN:389	-0.66	0.02	-3.69	0.07	4.19	0.06	0	0	-0.17	0.11
B	LYS:397	-0.66	0.03	-140.14	0.24	137.61	0.22	0	0	-3.19	0.33
B	PRO:398	-2.93	0.02	-1.79	0.02	3.24	0.02	0	0	-1.47	0.06
B	GLY:399	-1.08	0.02	0.22	0.02	0.48	0.01	0	0	-0.38	0.05
B	ILE:400	-4.71	0.03	-4.7	0.04	4.96	0.01	0	0	-4.44	0.07
B	PRO:401	-3.53	0.02	1.88	0.03	-0.5	0.03	0	0	-2.15	0.07
B	LYS:402	0.46	0.03	-162.48	0.18	156.18	0.15	0	0	-5.84	0.24
B	LYS:405	-1.08	0.03	-117.52	0.31	122.3	0.3	0	0	3.7	0.44
B	ASN:407	-1.42	0.03	-1.12	0.09	1.05	0.06	0	0	-1.49	0.12
B	GLY:408	-1.62	0.01	-3.89	0.05	3.66	0.03	0	0	-1.85	0.07
B	GLY:409	-1.34	0.01	-1.97	0.03	1.84	0.03	0	0	-1.47	0.06
B	GLY:410	-2.57	0.02	-5.6	0.07	6.65	0.03	0	0	-1.52	0.09
B	LYS:411	-0.87	0.03	-164.65	0.23	158.5	0.2	0	0	-7.02	0.31
B	ARG:412	-0.91	0.03	-98.75	0.24	98.78	0.21	0	0	-0.89	0.34
B	LYS:413	-1.33	0.02	-185.1	0.16	183.39	0.13	0	0	-3.04	0.22
B	ARG:414	-0.4	0.04	-118.23	0.15	116.54	0.1	0	0	-2.1	0.2
B	TRP:426	-0.44	0.03	-1.56	0.05	1.64	0.02	0	0	-0.35	0.09
B	SER:428	-0.7	0.02	-4.99	0.07	5.31	0.04	0	0	-0.37	0.1
B	LEU:468	-0.21	0.02	132.2	0.16	-129.57	0.14	0	0	2.43	0.22

*SEM: Standard Error of Mean

Table S4. Per-residue decomposition of the binding free energy for Col_E3 (chain B) in complex with Im3 (chain A), obtained in Run2 using the MM-PBSA method with energy values given in kcal/mol.

Chain	Residue	van der Waals		Electrostatics		Polar Solvation		Non-Polar Solvation		Total	
		Avg.	SEM	Avg.	SEM	Avg.	SEM	Avg.	SEM	Avg.	SEM
A	GLY:1	-0.14	0.03	59.92	0.23	-59.54	0.16	0	0	0.25	0.28
A	LEU:2	-0.68	0.02	1.11	0.03	-1.08	0.01	0	0	-0.65	0.06
A	ASP:5	0.79	0.03	-134.66	0.15	131.41	0.13	0	0	-2.46	0.21
A	THR:7	-0.76	0.02	-0.06	0.04	0.67	0.02	0	0	-0.15	0.07
A	PHE:9	-4.12	0.03	0.84	0.03	-0.15	0.01	0	0	-3.43	0.07
A	ASP:10	-0.24	0.03	-64.8	0.15	64.88	0.12	0	0	-0.16	0.2
A	GLU:14	-0.75	0.02	-102.1	0.11	102.1	0.1	0	0	-0.75	0.16
A	ASP:15	-0.14	0.03	-87.06	0.31	86.15	0.28	0	0	-1.05	0.43
A	PHE:16	-2.86	0.03	0.94	0.04	-0.11	0.03	0	0	-2.03	0.09
A	GLU:19	-0.51	0.02	-80.44	0.13	81.2	0.13	0	0	0.25	0.19
A	TYR:21	-0.97	0.03	3.76	0.05	-2.71	0.03	0	0	0.08	0.09
A	GLY:26	-0.15	0.02	-0.86	0.04	1.41	0.02	0	0	0.41	0.06
A	ASP:27	-0.58	0.03	-73.02	0.24	72.26	0.19	0	0	-1.34	0.31
A	ASP:28	-0.74	0.03	-49.57	0.18	50.4	0.15	0	0	0.09	0.24
A	GLY:29	-0.88	0.02	0.07	0.03	0.38	0.02	0	0	-0.43	0.06
A	MET:32	-1.07	0.02	0.19	0.02	-0.03	0.01	0	0	-0.91	0.06
A	GLU:33	-1.88	0.02	-68.28	0.25	70.12	0.23	0	0	-0.03	0.35
A	SER:34	-2.3	0.02	0.96	0.04	1.87	0.03	0	0	0.53	0.07
A	LEU:35	-0.82	0.02	-0.33	0.03	0.38	0.01	0	0	-0.77	0.06
A	GLY:36	-1.46	0.01	-2.19	0.03	2.95	0.02	0	0	-0.7	0.05
A	VAL:37	-1.11	0.02	0.69	0.02	-0.01	0.01	0	0	-0.43	0.06
A	PRO:38	-3.12	0.03	-3.57	0.02	4.31	0.01	0	0	-2.37	0.06
A	PHE:39	-4.95	0.03	-2.51	0.03	4.84	0.02	0	0	-2.62	0.08
A	LYS:40	-3.71	0.03	24.36	0.25	-23.35	0.21	0	0	-2.7	0.33
A	ASP:41	-1.84	0.03	-54.38	0.18	56.02	0.16	0	0	-0.21	0.24
A	ASN:42	-1.13	0.02	-8.34	0.04	8.75	0.02	0	0	-0.71	0.07
A	VAL:43	-2.91	0.02	1.59	0.02	1.57	0.01	0	0	0.24	0.06
A	ASN:44	-4.17	0.02	-6.01	0.04	8.6	0.02	0	0	-1.58	0.07
A	ASN:45	-4.2	0.02	-5.75	0.04	8.6	0.02	0	0	-1.36	0.07
A	GLY:46	-2.21	0.02	0.56	0.03	0.75	0.02	0	0	-0.91	0.06
A	CYS:47	-2.65	0.02	-7.12	0.03	6.28	0.02	0	0	-3.5	0.06
A	PHE:48	-2.53	0.03	0.57	0.03	0.03	0.02	0	0	-1.92	0.07
A	ASP:49	-0.12	0.03	-100.07	0.24	99.24	0.23	0	0	-0.95	0.33
A	ILE:51	-0.39	0.02	0.48	0.03	-0.43	0.02	0	0	-0.33	0.07
A	ALA:52	-0.91	0.02	1.24	0.03	-0.95	0.02	0	0	-0.62	0.06
A	GLU:53	-3.25	0.02	-49.13	0.13	55.32	0.11	0	0	2.94	0.18
A	TRP:54	-0.78	0.03	0.01	0.03	0.06	0.01	0	0	-0.71	0.08
A	PRO:56	-3.37	0.02	-2.5	0.02	2.31	0.01	0	0	-3.56	0.06
A	LEU:57	-3.55	0.02	1.08	0.02	0.24	0.01	0	0	-2.23	0.06
A	GLN:59	-1.88	0.03	-1.48	0.04	2.17	0.02	0	0	-1.19	0.08
A	PRO:60	-3.04	0.02	-0.44	0.02	0.56	0.02	0	0	-2.93	0.06
A	TYR:61	-1.26	0.03	-0.51	0.02	1.83	0.02	0	0	0.06	0.07
A	GLN:65	-1.49	0.02	-2.6	0.06	4.04	0.04	0	0	-0.05	0.09
A	ASP:67	-0.16	0.03	-49.17	0.12	48.93	0.1	0	0	-0.4	0.17
A	ILE:68	-0.2	0.03	-0.3	0.04	0.27	0.03	0	0	-0.23	0.08
A	SER:69	-0.11	0.02	-0.31	0.07	0.28	0.06	0	0	-0.13	0.1
A	GLU:72	-0.62	0.03	-74.78	0.26	74.62	0.22	0	0	-0.78	0.34
A	PHE:74	-3.15	0.03	1.03	0.02	-0.02	0.01	0	0	-2.14	0.07
A	VAL:75	-0.24	0.02	-1.08	0.02	1.03	0.01	0	0	-0.3	0.06
A	SER:76	-0.62	0.02	0.15	0.04	0.19	0.01	0	0	-0.27	0.06
A	PHE:77	-0.43	0.03	-0.76	0.03	0.76	0.01	0	0	-0.43	0.07
A	ASP:78	0.5	0.03	-106.23	0.3	101.91	0.25	0	0	-3.82	0.39
A	TYR:79	-3.39	0.04	-1.66	0.06	3.85	0.03	0	0	-1.21	0.1
A	ARG:80	-1.44	0.04	66.64	0.24	-63.99	0.18	0	0	1.21	0.31
A	ASP:81	-0.04	0.03	-72.27	0.2	70.9	0.18	0	0	-1.42	0.27
B	PHE:46	-3.96	0.04	-6.31	0.03	6.93	0.02	0	0	-3.33	0.08
B	GLY:47	-0.92	0.01	-4.34	0.02	3.59	0.01	0	0	-1.67	0.05
B	LEU:48	-0.88	0.02	0.22	0.02	-0.11	0.01	0	0	-0.77	0.06
B	ALA:94	-0.48	0.02	-1.47	0.03	1.61	0.02	0	0	-0.33	0.06
B	THR:95	-1.41	0.03	-0.64	0.04	1.27	0.02	0	0	-0.79	0.07
B	ASN:97	-0.78	0.02	-0.64	0.07	1.27	0.05	0	0	-0.15	0.1
B	ARG:101	-1.63	0.03	-97.49	0.09	103.02	0.09	0	0	3.9	0.15
B	VAL:119	-1.75	0.03	-2.69	0.02	2.7	0.01	0	0	-1.74	0.06
B	PRO:120	-2.99	0.02	1.23	0.02	2.08	0.01	0	0	0.31	0.06
B	MET:121	-2.57	0.02	-2.16	0.02	2.59	0.01	0	0	-2.14	0.06
B	SER:122	-2.44	0.02	-0.7	0.04	2.51	0.02	0	0	-0.63	0.07
B	VAL:123	-0.76	0.03	0.11	0.02	-0.02	0.01	0	0	-0.66	0.06
B	PRO:124	-0.41	0.02	-2.02	0.02	1.96	0.01	0	0	-0.47	0.06

B	PRO:142	-0.24	0.02	-1.3	0.03	1.33	0.02	0	0	-0.21	0.06
B	GLY:143	-0.09	0.01	-0.64	0.02	0.65	0.02	0	0	-0.09	0.05
B	PHE:178	-0.61	0.03	2.99	0.03	-2.7	0.02	0	0	-0.32	0.08
B	THR:179	-0.53	0.03	0.13	0.04	1.27	0.02	0	0	0.87	0.07
B	GLN:180	-0.99	0.02	3.02	0.04	-0.96	0.03	0	0	1.07	0.08
B	GLY:181	-0.69	0.02	1.97	0.03	-0.61	0.02	0	0	0.67	0.05
B	GLY:182	-0.79	0.01	-0.07	0.05	0.21	0.03	0	0	-0.64	0.07
B	THR:184	-0.24	0.03	0.5	0.05	-0.45	0.02	0	0	-0.19	0.08
B	ARG:185	-1.24	0.03	-103.18	0.17	102.87	0.16	0	0	-1.55	0.25
B	ASP:186	-0.27	0.04	91.19	0.09	-89.01	0.07	0	0	1.91	0.13
B	ARG:223	-0.8	0.03	-117.46	0.14	116.5	0.13	0	0	-1.75	0.2
B	GLU:226	-0.57	0.03	83.18	0.23	-81.29	0.22	0	0	1.33	0.32
B	TRP:227	-2.02	0.04	0.18	0.05	0.37	0.03	0	0	-1.47	0.1
B	ALA:229	-0.33	0.02	-0.5	0.03	0.63	0.02	0	0	-0.2	0.06
B	THR:230	-1.75	0.03	-0.98	0.04	2.42	0.03	0	0	-0.31	0.08
B	HIS:231	-0.93	0.03	0.46	0.1	1.23	0.07	0	0	0.76	0.13
B	LYS:372	-0.3	0.03	-86.18	0.17	85.18	0.15	0	0	-1.31	0.23
B	GLY:373	-0.33	0.02	-1.64	0.03	1.69	0.01	0	0	-0.28	0.05
B	PHE:374	-7.5	0.03	-1.84	0.03	5.17	0.02	0	0	-4.18	0.08
B	LYS:375	-1.11	0.03	-156.45	0.28	153.28	0.24	0	0	-4.28	0.38
B	TYR:377	-4.36	0.03	-0.62	0.06	3.33	0.04	0	0	-1.65	0.1
B	GLY:378	-1.75	0.01	-2.34	0.02	3.23	0.01	0	0	-0.86	0.05
B	HIS:379	-0.91	0.02	-3.65	0.05	3.57	0.04	0	0	-0.98	0.08
B	ASP:380	-0.57	0.03	92.23	0.16	-89.27	0.16	0	0	2.39	0.23
B	TYR:381	-3.34	0.03	-9.12	0.05	6.23	0.03	0	0	-6.23	0.09
B	HIS:382	-0.81	0.02	3.45	0.05	-2.52	0.03	0	0	0.13	0.08
B	PRO:383	-2.57	0.02	-4.43	0.02	4.8	0.01	0	0	-2.2	0.06
B	ALA:384	-0.46	0.02	-0.35	0.05	0.05	0.03	0	0	-0.76	0.08
B	LYS:386	-0.43	0.03	-183.45	0.2	181.15	0.16	0	0	-2.73	0.26
B	THR:387	-0.29	0.02	-5.75	0.05	5.92	0.04	0	0	-0.13	0.09
B	GLU:388	-0.11	0.02	107.56	0.18	-106.03	0.17	0	0	1.42	0.25
B	LYS:397	-0.59	0.03	-127.91	0.27	125.94	0.24	0	0	-2.56	0.37
B	PRO:398	-2.81	0.02	-1.65	0.03	2.89	0.03	0	0	-1.57	0.07
B	GLY:399	-1.07	0.02	0.01	0.02	0.78	0.01	0	0	-0.28	0.05
B	ILE:400	-4.85	0.03	-4.54	0.02	4.76	0.01	0	0	-4.63	0.07
B	PRO:401	-3.48	0.02	2.32	0.03	-0.99	0.02	0	0	-2.15	0.06
B	LYS:402	0.53	0.03	-163.34	0.13	156.89	0.11	0	0	-5.92	0.18
B	LYS:405	-3.45	0.03	-129.37	0.14	133.7	0.12	0	0	0.88	0.2
B	GLN:406	-0.41	0.03	5.04	0.07	-4.88	0.05	0	0	-0.25	0.11
B	ASN:407	-1.16	0.03	1.14	0.08	-0.63	0.06	0	0	-0.65	0.11
B	GLY:408	-0.31	0.01	1.16	0.03	-1.23	0.02	0	0	-0.37	0.06
B	GLY:409	-0.71	0.02	-0.45	0.05	0.17	0.02	0	0	-0.99	0.07
B	GLY:410	-1.54	0.01	-6.44	0.03	6.8	0.02	0	0	-1.18	0.06
B	LYS:411	-1.07	0.03	-154.7	0.3	150.65	0.26	0	0	-5.11	0.41
B	ARG:412	-1.04	0.03	-100.55	0.13	100.24	0.1	0	0	-1.35	0.18
B	LYS:413	-1.29	0.02	-189.17	0.12	185.76	0.1	0	0	-4.7	0.17
B	THR:416	-0.16	0.03	0.13	0.06	-0.16	0.04	0	0	-0.19	0.09
B	TRP:426	-0.38	0.03	-1.49	0.03	1.42	0.02	0	0	-0.45	0.08
B	SER:428	-0.58	0.02	-5.27	0.06	5.01	0.04	0	0	-0.84	0.09
B	GLN:429	-0.24	0.02	-0.6	0.09	0.7	0.08	0	0	-0.15	0.13
B	LEU:468	-0.2	0.03	132.7	0.14	-130.2	0.13	0	0	2.3	0.2

*SEM: Standard Error of Mean

Table S5. Per-residue decomposition of the binding free energy for Col_E8 (chain B) in complex with Im8 (chain A), obtained in Run1 using the MM-PBSA method with energy values given in kcal/mol.

Chain	Residue	van der Waals		Electrostatics		Polar Solvation		Non-Polar Solvation		Total	
		Avg.	SEM	Avg.	SEM	Avg.	SEM	Avg.	SEM	Avg.	SEM
A	GLU:2	-0.03	0.02	-39.28	0.24	39	0.22	0	0	-0.31	0.33
A	ILE:22	-0.46	0.03	-3.44	0.03	3.23	0.02	0	0	-0.67	0.07
A	ILE:23	-1.93	0.03	-3.77	0.02	5.64	0.01	0	0	-0.06	0.07
A	ASN:24	-1.15	0.02	-6.4	0.05	7.29	0.04	0	0	-0.26	0.08
A	CYS:25	-2.95	0.02	-0.17	0.03	1.36	0.02	0	0	-1.75	0.06
A	GLU:26	-0.25	0.02	-68.3	0.12	67.27	0.13	0	0	-1.28	0.19
A	GLY:27	-0.15	0.01	-3.77	0.04	3.79	0.03	0	0	-0.14	0.06
A	ASP:28	-0.23	0.02	-63.06	0.14	62.31	0.14	0	0	-0.98	0.21
A	GLU:29	-0.86	0.03	-105.85	0.24	105.64	0.21	0	0	-1.08	0.33
A	LYS:30	-0.36	0.03	57.25	0.2	-56.79	0.18	0	0	0.1	0.27
A	GLN:32	-1.65	0.02	-9.52	0.05	8.62	0.03	0	0	-2.55	0.08
A	ASP:33	-0.12	0.03	-128.17	0.14	123.61	0.14	0	0	-4.69	0.2
A	LEU:36	-2.19	0.03	4.19	0.02	-3.38	0.01	0	0	-1.39	0.06
A	GLU:37	-0.97	0.03	-75.6	0.15	75.26	0.13	0	0	-1.31	0.21
A	ILE:40	-1.01	0.03	3.06	0.02	-2.67	0.01	0	0	-0.62	0.07
A	PRO:46	-0.47	0.02	-3.54	0.03	4.06	0.02	0	0	0.05	0.06
A	SER:47	-0.76	0.02	-2.46	0.05	3.1	0.04	0	0	-0.12	0.08
A	GLY:48	-0.49	0.02	-0.41	0.03	0.57	0.02	0	0	-0.33	0.05
A	SER:49	-1.46	0.02	0.38	0.06	1.66	0.02	0	0	0.59	0.08
A	ASP:50	-1.37	0.03	-106.97	0.13	115.54	0.12	0	0	7.2	0.19
A	ILE:52	-0.6	0.03	-0.66	0.03	1.76	0.01	0	0	0.5	0.07
A	TYR:53	-5.58	0.03	-3.46	0.04	6.55	0.02	0	0	-2.48	0.08
A	TYR:54	-5.12	0.04	-4.36	0.03	5.14	0.02	0	0	-4.34	0.08
A	PRO:55	-0.8	0.02	-0.37	0.02	0.54	0.02	0	0	-0.63	0.07
A	GLU:56	-0.11	0.02	-73.97	0.16	73.27	0.15	0	0	-0.81	0.23
A	GLY:61	-0.21	0.02	-1.46	0.02	1.49	0.01	0	0	-0.17	0.05
B	PHE:46	-0.03	0.04	-0.61	0.08	0.61	0.05	0	0	-0.03	0.12
B	LYS:93	-0.03	0.02	-41.76	0.27	41.03	0.24	0	0	-0.75	0.37
B	THR:95	-0.02	0.02	-0.23	0.05	0.22	0.04	0	0	-0.02	0.08
B	VAL:119	-0.01	0.03	-0.88	0.02	0.86	0.02	0	0	-0.03	0.07
B	ARG:418	0.13	0.03	-103.9	0.23	100.4	0.17	0	0	-3.37	0.3
B	GLN:434	-0.1	0.03	2.14	0.09	-2	0.09	0	0	0.03	0.14
B	PHE:435	-0.37	0.03	-1.77	0.04	2.21	0.03	0	0	0.07	0.09
B	ASN:436	-2.44	0.03	-3.73	0.09	4.88	0.07	0	0	-1.28	0.13
B	PRO:437	-1.15	0.02	-0.38	0.02	0.51	0.02	0	0	-1.02	0.07
B	GLY:438	-1.84	0.01	-0.45	0.03	2.76	0.02	0	0	0.47	0.06
B	ASN:439	-2.13	0.02	0.7	0.04	0.65	0.02	0	0	-0.78	0.07
B	LYS:441	-2.31	0.02	-106.62	0.17	106.49	0.16	0	0	-2.44	0.24
B	ARG:442	-2.46	0.03	-136.54	0.13	142.64	0.11	0	0	3.64	0.19
B	GLN:445	-0.91	0.02	-2.2	0.07	2.7	0.06	0	0	-0.42	0.11
B	LEU:447	-1.57	0.02	-3.38	0.03	3.4	0.01	0	0	-1.56	0.06
B	ALA:448	-0.15	0.02	3.52	0.03	-4.08	0.01	0	0	-0.71	0.06
B	PRO:449	-0.37	0.02	0.28	0.02	0.19	0.01	0	0	0.1	0.06
B	ARG:450	-2.54	0.03	-157.04	0.12	157.97	0.09	0	0	-1.62	0.17
B	ALA:451	-1.06	0.02	2.85	0.03	-1.56	0.02	0	0	0.24	0.05
B	ARG:452	-3.83	0.03	-126.47	0.29	123.61	0.25	0	0	-6.68	0.39
B	ASN:453	-2.56	0.03	-12.55	0.08	10.44	0.06	0	0	-4.67	0.12
B	LYS:454	-0.4	0.02	-92.43	0.27	91.27	0.23	0	0	-1.55	0.36
B	THR:456	-0.32	0.02	-1.49	0.07	1.68	0.05	0	0	-0.12	0.1
B	GLY:458	-0.16	0.01	3.31	0.04	-3.3	0.03	0	0	-0.15	0.06
B	GLY:459	-0.49	0.01	3.58	0.03	-2.82	0.02	0	0	0.26	0.05
B	ARG:461	-3.57	0.03	-126.73	0.16	130.15	0.14	0	0	-0.15	0.23
B	SER:462	-0.28	0.02	0.23	0.04	-0.72	0.03	0	0	-0.78	0.07
B	PHE:463	-0.41	0.03	1.99	0.03	-1.74	0.02	0	0	-0.17	0.08

*SEM: Standard Error of Mean

Table S6. Per-residue decomposition of the binding free energy for Col_E8 (chain B) in complex with Im8 (chain A), obtained in Run2 using the MM-PBSA method with energy values given in kcal/mol.

Chain	Residue	van der Waals		Electrostatics		Polar Solvation		Non-Ploar Solvation		Total	
		Avg.	SEM	Avg.	SEM	Avg.	SEM	Avg.	SEM	Avg.	SEM
A	CYS:25	-3.29	0.02	0.63	0.04	0.62	0.02	0	0	-2.04	0.07
A	GLU:26	-0.25	0.02	-65.3	0.11	64.38	0.11	0	0	-1.17	0.17
A	GLY:27	-0.14	0.01	-3.9	0.04	3.83	0.03	0	0	-0.21	0.06
A	ASP:28	-0.28	0.02	-57.4	0.16	56.83	0.15	0	0	-0.85	0.22
A	GLU:29	-0.6	0.03	-108.51	0.24	108.86	0.22	0	0	-0.26	0.33
A	LYS:30	-1.36	0.03	55.6	0.34	-52.9	0.31	0	0	1.34	0.47
A	GLN:32	-1.43	0.03	-8.94	0.05	8.35	0.03	0	0	-2.02	0.08
A	ASP:33	-0.2	0.03	-115.08	0.28	111.23	0.25	0	0	-4.05	0.38
A	LEU:36	-2.08	0.02	3.5	0.02	-3.06	0.01	0	0	-1.64	0.06
A	GLU:37	-0.68	0.03	-85.7	0.23	86.66	0.21	0	0	0.28	0.32
A	ILE:40	-1.1	0.03	2.87	0.03	-2.53	0.01	0	0	-0.76	0.07
A	HIS:45	-0.15	0.03	-3.34	0.05	3.17	0.03	0	0	-0.32	0.08
A	PRO:46	-0.93	0.02	-3.56	0.02	4.82	0.02	0	0	0.33	0.06
A	SER:47	-0.89	0.02	-1.38	0.05	2.36	0.04	0	0	0.08	0.08
A	GLY:48	-0.27	0.02	-0.42	0.02	0.58	0.01	0	0	-0.12	0.05
A	SER:49	-1.59	0.02	-0.68	0.04	2.98	0.02	0	0	0.71	0.07
A	ASP:50	-1.33	0.03	-107.09	0.12	117.53	0.12	0	0	9.12	0.18
A	ILE:52	-0.71	0.02	-0.8	0.02	1.94	0.01	0	0	0.43	0.06
A	TYR:53	-5.99	0.03	-2.53	0.03	6.58	0.02	0	0	-1.93	0.08
A	TYR:54	-4.84	0.04	-3.79	0.03	4.56	0.02	0	0	-4.07	0.08
A	PRO:55	-0.73	0.02	-0.48	0.02	0.62	0.02	0	0	-0.58	0.06
A	GLU:56	-0.12	0.02	-73	0.15	72.45	0.14	0	0	-0.67	0.21
A	ASP:60	-0.17	0.03	-58.74	0.11	58.17	0.11	0	0	-0.74	0.16
A	GLY:61	-0.2	0.02	-1.23	0.02	1.27	0.01	0	0	-0.16	0.04
B	PHE:46	-0.34	0.04	-1.17	0.07	1.22	0.05	0	0	-0.29	0.11
B	LYS:93	0.05	0.02	-49.1	0.24	48.09	0.23	0	0	-0.96	0.34
B	VAL:119	-0.05	0.04	-0.86	0.02	0.85	0.02	0	0	-0.06	0.07
B	PRO:120	-0.14	0.02	1.12	0.02	-1.03	0.02	0	0	-0.04	0.07
B	PRO:124	-0.03	0.02	-0.5	0.02	0.49	0.01	0	0	-0.05	0.06
B	PRO:142	-0.02	0.02	0.08	0.04	-0.05	0.02	0	0	0.01	0.07
B	ARG:418	0.94	0.04	-111.39	0.25	103.85	0.2	0	0	-6.6	0.33
B	GLN:434	-0.09	0.03	2.15	0.08	-2.06	0.06	0	0	0	0.11
B	PHE:435	-0.32	0.03	-1.69	0.04	1.87	0.03	0	0	-0.14	0.09
B	ASN:436	-2.55	0.03	-3.43	0.04	4.47	0.02	0	0	-1.51	0.08
B	PRO:437	-0.73	0.02	0.01	0.02	0.06	0.02	0	0	-0.66	0.07
B	GLY:438	-1.57	0.02	-0.33	0.03	1.94	0.01	0	0	0.04	0.05
B	ASN:439	-2.06	0.02	1.06	0.04	-0.53	0.02	0	0	-1.53	0.07
B	LYS:441	-2.38	0.02	-104.58	0.18	104.08	0.18	0	0	-2.89	0.26
B	ARG:442	-2.72	0.03	-133.99	0.12	140.97	0.09	0	0	4.26	0.17
B	GLN:445	-1.03	0.02	-3.41	0.09	3.57	0.07	0	0	-0.87	0.13
B	LEU:447	-1.97	0.02	-3.76	0.03	3.77	0.01	0	0	-1.96	0.06
B	ALA:448	-0.2	0.02	3.22	0.03	-3.77	0.01	0	0	-0.75	0.06
B	PRO:449	-0.37	0.02	0.54	0.03	-0.18	0.01	0	0	-0.02	0.06
B	ARG:450	-2.55	0.03	-152.7	0.12	153.11	0.09	0	0	-2.14	0.17
B	ALA:451	-1.19	0.02	2.93	0.03	-0.67	0.02	0	0	1.07	0.06
B	ARG:452	-3.84	0.03	-122.59	0.24	120.53	0.21	0	0	-5.91	0.33
B	ASN:453	-1.73	0.03	-12.45	0.09	9.47	0.07	0	0	-4.71	0.13
B	LYS:454	-0.3	0.02	-86.13	0.29	85.06	0.25	0	0	-1.37	0.39
B	THR:456	-0.3	0.03	-0.88	0.07	0.6	0.04	0	0	-0.58	0.09
B	GLY:458	-0.58	0.02	0.2	0.04	-0.38	0.03	0	0	-0.76	0.07
B	GLY:459	-1.34	0.01	2.18	0.05	-0.63	0.04	0	0	0.21	0.07
B	ARG:461	-3.56	0.03	-114.9	0.14	118.47	0.11	0	0	0.01	0.2
B	SER:462	-0.36	0.02	0.64	0.05	-1.05	0.03	0	0	-0.77	0.08
B	PHE:463	-0.43	0.03	2.43	0.03	-2.19	0.02	0	0	-0.19	0.07

*SEM: Standard Error of Mean
